# Response to high dose ipilimumab plus temozolomide after progression on standard or low dose ipilimumab in advanced melanoma: a retrospective analysis

**DOI:** 10.21203/rs.3.rs-2997157/v1

**Published:** 2023-06-01

**Authors:** Julie Williamson, Muhammad Zaki Hidayatullah Fadlullah, Magdalena Kovacsovics-Bankowski, Berit Gibson, Umang Swami, Alyssa Erickson-Wayman, Debra Jamison, Dan Sageser, Joanne Jeter, Tawnya Bowles, Donald M. Cannon, Ben Haaland, Joyce D Schroeder, David Nix, Aaron Atkinson, John Hyngstrom, Jordan McPherson, Aik-Choon Tan, Siwen Hu-Lieskovan

**Affiliations:** University of Utah; Huntsman Cancer Institute; Huntsman Cancer Institute; Huntsman Cancer Institute; Huntsman Cancer Institute; Huntsman Cancer Institute; Huntsman Cancer Institute; Huntsman Cancer Institute; Huntsman Cancer Institute; Intermountain Health; Huntsman Cancer Institute; Huntsman Cancer Institute; Huntsman Cancer Institute; Huntsman Cancer Institute; Huntsman Cancer Institute; Huntsman Cancer Institute; Huntsman Cancer Institute; Huntsman Cancer Institute; University of Utah

**Keywords:** Advanced melanoma, immune checkpoint inhibitors, translational research

## Abstract

**Background:**

Despite advancements in checkpoint inhibitor-based immunotherapy, patients with advanced melanoma who have progressed on standard dose ipilimumab (Ipi) + nivolumab continue to have poor prognosis. Several studies support a dose-response activity of Ipi, and one promising combination is Ipi 10mg/kg (Ipi10) + temozolomide (TMZ).

**Methods:**

We performed a retrospective cohort analysis of patients with advanced melanoma treated with Ipi10+TMZ in the immunotherapy refractory/resistant setting (n = 6), using similar patients treated with Ipi3+TMZ (n = 6) as comparison. Molecular profiling by whole exome sequencing (WES) and RNA-seq of tumors harvested through one responder’s treatment was performed.

**Results:**

With a median follow up of 119 days, patients treated with Ipi10+TMZ had statistically significant longer median progression free survival of 144.5 days (range 27–219) vs 44 (26–75) in Ipi3+TMZ, p=0.04, and a trend for longer median overall survival of 154.5 days (27–537) vs 89.5 (26–548). All patients in the Ipi10 cohort had progressed on prior Ipi+Nivo. WES revealed only 12 shared somatic mutations including BRAF V600E. RNA-seq showed enrichment of inflammatory signatures, including interferon responses in metastatic lesions after standard dose Ipi + nivo and Ipi10 + TMZ compared to the primary tumor, and downregulated negative immune regulators including Wnt and TGFb signaling.

**Conclusion:**

Ipi10+TMZ demonstrated efficacy including dramatic responses in patients with advanced melanoma refractory to prior Ipi + anti-PD1, even with CNS metastases. Molecular data suggest a potential threshold of Ipi dose for activation of sufficient anti-tumor immune response, and higher dose Ipi is required for some patients.

## Introduction

The advent of checkpoint inhibitor-based immunotherapy has drastically changed the treatment and prognosis of advanced melanoma in the past decade. The combination of ipilimumab (Ipi) 3 mg/kg plus nivolumab 1 mg/kg has become the standard of care, achieving 52% overall survival and 36% progression free survival at 5 years.[1] However, this leaves an unmet need for the majority of patients who still progress and those who do not survive past 5 years. New immunotherapy agents are entering the market, with relatlimab, an antibody against LAG-3, the latest to gain FDA approval for advanced melanoma. However, the complete response rate to relatlimab plus nivolumab in untreated advanced melanoma was 16.3% and the partial response rate was 26.8%, which unfortunately still leaves many with progression of their disease. [2, 3] Patients with symptomatic brain metastases continue to fare poorly, with 36% overall survival and 19% overall survival at 3 years on combination Ipi and nivolumab.[4] Dosing of this immunotherapy combination has been studied, although primarily within the lens of safety profiles. Flipped dosing with Ipi 1 mg/kg plus nivolumab 3 mg/kg had significantly reduced rates of severe adverse effects while maintaining similar progression free and overall survival at 3 years, so higher doses are not routinely prescribed or studied. [5] However, before combination therapy became standard of care, an Italian study demonstrated a significant improvement in overall survival with Ipi 10 mg/kg compared to 3 mg/kg. [6] Additionally, using standard dose Ipi following progression on low-dose Ipi has shown clinical activity in patients with metastatic melanoma.[7] This evidence supports the dose-response activity of Ipi.

One promising combination for these patients is Ipi 10 mg/kg plus temozolomide (TMZ). TMZ works by depleting regulatory T cells and suppressing their function, and it may enhance the antitumor activity of Ipi when dosed together. [8, 9] One study found a response rate of 31% using this combination in immunotherapy naïve patients with advanced melanoma, whereas Ipi 3 mg/kg and 10mg/kg alone had response rates of 12% and 15%, respectively. [6, 10, 11] TMZ crosses the blood brain barrier and therefore may improve treatment efficacy in patients with brain metastases.

The aim of this retrospective case series is to describe outcomes for patients with advanced melanoma who were treated with high dose (10 mg/kg) Ipi plus TMZ in the immunotherapy refractory/resistant setting, using a cohort of similar patients treated with standard dose (3 mg/kg) Ipi plus TMZ as comparison. Two patients with CNS involvement demonstrated an extraordinary response to Ipi 10 mg/kg plus TMZ despite progressing on regular or low dose IPI plus nivolumab and other treatments previously.

## Methods

Retrospective chart review was performed to identify patients with advanced melanoma treated with Ipi plus TMZ at Huntsman Cancer Institute from June 1, 2014 to June 30, 2022, under University of Utah approved IRB# 00138167. Data was extracted from a treatment plan search activity within EPIC. Two cohorts were identified; cohort A (n = 6 patients) included those treated with high dose Ipi (10 mg/kg) plus TMZ, and cohort B (n = 6 patients) included those treated with standard dose Ipi (3 mg/kg) plus TMZ. Ipi was dosed on day 1 every 3 weeks for 4 cycles. TMZ was dosed as 200mg/m^2^ on days 1–4 every 3 weeks for 4 cycles.

### Sample collection and preparation

Archival specimen collected from one patient included a punch biopsy of the primary site, an ultrasound-guided core needle biopsy of the liver, and a core needle biopsy of a lesion on the back. All samples were placed in formalin and made into FFPE blocks. The samples were stained with Melan-A and SOX10 and had clinical pathology review to confirm diagnosis Unstained slides were submitted to Tempus for sequencing.

### Whole exome sequencing

Whole exome sequencing of the samples was performed at the Tempus Lab, Inc. (Chicago, IL.) Fastq were aligned to the human reference genome GRCh38 using BWA [12]. Next, PCR duplicates were removed, and somatic variants (INDELs and SNVs) were called using Illumina’s Manta and Strelka2 software [13]. The alignment, variant calling, variant annotations were integrated in containerized Snakemake workflows that are publicly available at https://github.com/HuntsmanCancerInstitute/Workflows. Next the mutations were filtered to retain deleterious mutations; that is if a mutation falls into protein coding genes and is considered ‘deleterious’ according to Sorting Intolerant From Tolerant (SIFT) and ‘probably damaging’ or ‘possibly damaging’ according to Polymorphism Phenotyping version 2 (PolyPhen-2). Variant annotations were done using the Ensembl Variant Effect Predictor tool [14]. The deleterious mutations list is included as Supplemental Table 1. Tumor Mutational Burden (TMB) calculation was based on the number of deleterious mutations divided by the length of human exons (38Mb). Phylogenetic tree was constructed based on the Neighbor joining method implemented in the R phangorn package [15] Pathway enrichment analysis were based on the web-based tool Enrichr [16] using the Hallmark 2020 pathway.

### RNA-sequencing

Samples were sequenced by the Tempus Lab, Inc. (Chicago, IL.). RNA-seq fastq were aligned with STAR aligner [17] and gene expression were quantified using RSEM [18]. To determine enriched gene signatures in the samples, Single Sample Gene Set Enrichment Analysis (ssGSEA) was conducted using the curated Hallmark gene sets (h.all.v2022.1.Hs.symbols.gmt). The R package GSVA [19] was used to calculate the normalized ssGSEA pathway score and the normalized results was scaled and displayed as heatmap.

## Results

### Case study 1:

**Figures 1:**
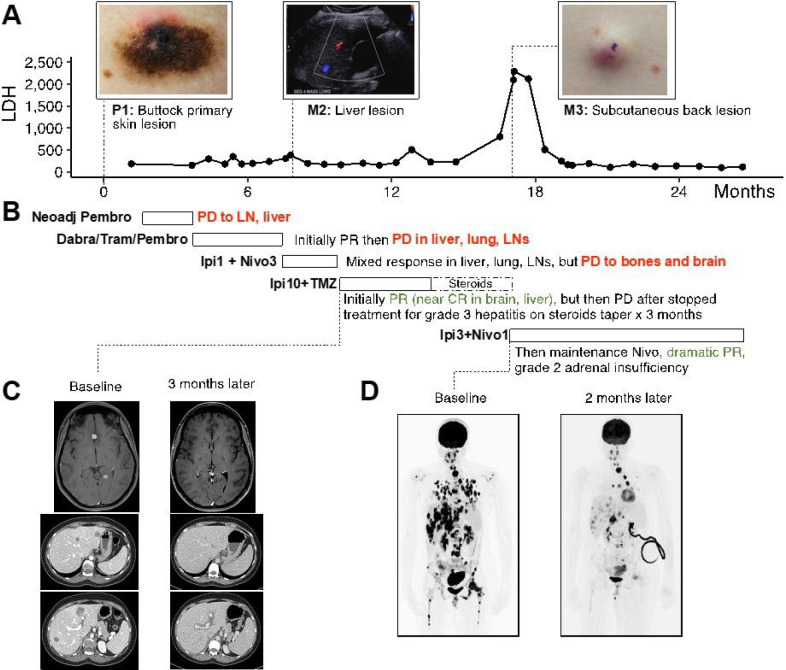
Timeline of patient 10A’s treatment. A) Lactate Dehydrogenase (LDH) is plotted against time. Point of time when specimens were collected for next generation sequencing profiling is indicated as primary (P) and metastatic (M). B) Timeline describing treatment regime. C) Brain MRI revealing two metastatic lesions prior to first dose of Ipi10 plus TMZ, one at the interhemispheric fissure between the inferior frontal lobe and one at the left paramedian occipital lobe; this was the first evidence of CNS involvement. Brain MRI 12.5 weeks later, after four cycles of Ipi 10 mg/kg plus TMZ, revealed complete resolution of the anterior nodule and posttreatment changes and completely resolved perilesional vasogenic edema of the occipital nodule. D) PET scan 4 months after initiating high dose steroids and holding treatment for liver toxicity from Ipi10 plus TMZ showed diffuse disease progression. PET scan 9.5 weeks later, after receiving 3 cycles standard dose Ipi plus nivolumab showed dramatic partial response.

A 31-year-old female (patient 10A) was initially diagnosed with stage IIIc BRAF V600E positive nodular melanoma to the skin of buttock. She was started on neoadjuvant pembrolizumab 200mg IV every 3 weeks in a clinical trial setting. After 3 cycles, she was found to have disease progression to stage IV (pT4b, N3c, M1c) with lymph node and liver metastases by the time of resection. She was then started on dabrafenib, trametinib, and pembrolizumab for four months. Pembrolizumab was intermittently held due to toxicity. She initially had a partial response but then had progression of disease in her liver, lungs, and lymph nodes. She was started on flipped dose Ipi (1 mg/kg) plus nivolumab (3 mg/kg) due to previous toxicity with pembrolizumab and tolerated 3 cycles. She had mixed response in her liver, stable disease in the lung and lymph nodes, but had progression to her bones and brain with new spine and femur lesions and two new 9 mm lesions in the left frontal and left occipital lobes. Her therapy was ultimately switched to high dose Ipi (10mg/kg) plus TMZ, and she received stereotactic radiosurgery to the brain lesions. Her subsequent scans showed decreased brain lesions and ultimately, she had complete resolution of her brain metastases and no new lesions by 3 months of treatment. After 4 cycles, she developed grade 3 hepatitis and was treated with high dose steroids with an extended period of steroid taper. While further treatment was held, she developed relapsed disease with new spinal, pulmonary, and liver lesions 5 months from the last dose of Ipi. Her lactate dehydrogenase (LDH) also peaked at this time to 2294. She was treated with standard dose Ipi (3mg/kg) plus nivolumab for 4 cycles followed by maintenance nivolumab and had another dramatic partial response (Figure 1) that is durable (no progression with 10 months of follow up now). At 17 months since high-dose Ipi was initiated, MRI brain still revealed no evidence of CNS disease. Whole body CT scan showed continued partial response except one left ovarian mass that has increased in size from 4.6cm to 5.4cm. She was taken to the operating room and underwent surgical metastatectomy of this ovarian mass. Pathology analysis revealed extensive tumor necrosis and histiocytic inflammation but no viable tumor was identified.

**Figure 2:**
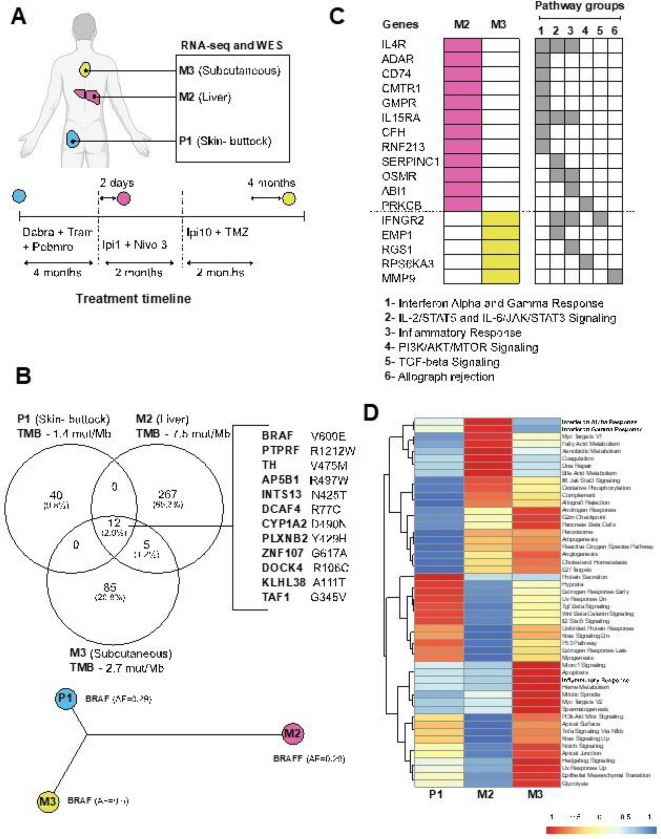
Exome and RNA-seq sequencing of tumors. A) Tumor samples collected at different anatomical sites from the patient, labelled P (primary) and M (metastasis). B) Top: The overlap of somatic mutations found in the tumor, with the tumor mutational burden (TMB) value (number of mutations/Mb) presented under each sample. Bottom: Construction of phylogenetic tree based on somatic mutations considered deleterious and damaging. The allele frequency (AF) of BRAF V600E is depicted. C) Enriched pathways of the genes uniquely mutated in samples M2 and M3. D) Heatmap of the ‘Hallmark Pathways’ showing the normalized result of single sample gene set enrichment analysis.

To further understand patient 10A’s treatment response, we analyzed molecular profiling by RNA-seq and whole-exome sequencing (WES) of the tumors harvested throughout her treatment, including the surgical specimen at the initial site of diagnosis (P1, harvested from skin of the buttock) and subsequent site of metastasis to the liver (M2) 2 days after first dose of low dose Ipi plus nivolumab and prior to the Ipi10 plus TMZ treatment, and to the subcutaneous soft tissue in the back (M3) after disease progression to Ipi10 plus TMZ and prior to subsequent Ipi 3 plus nivolumab (Figure 1A and 2A). WES revealed only 12 shared somatic mutations among these tumors (Figure 2B), including the BRAF V600E mutation, and other genes important in defining melanoma and continued activation of the MAPK pathway. Calculations of tumor mutational burden (TMB) indicates the liver metastasis (M2) has higher TMB 7.5 mutations/Mb than the primary tumor (P1) with 1.4 mutations/Mb and the later soft tissue metastasis (M3) with 2.7 mutations/Mb. We focused on the genes mutated in the immune- and inflammation-related pathways from the Hallmark gene sets, and found that the interferon alpha and gamma response pathways were enriched in the genes exclusively mutated in the M2 sample (Figure 2C). Interestingly, IFNGR2 is exclusively mutated in M3, which may play a role in altering the response to immunotherapy. To better understand the transcriptome changes during treatment, single sample gene set enrichment analysis (ssGSEA) was performed on the RNA-seq of these tumors. ssGSEA of the liver metastasis (M2) showed an enrichment of immune-related gene sets including interferon alpha and gamma responses (Figure 2D). M3, when compared to P1, is also enriched with the inflammatory response gene set. On the other hand, pathways shown to be negative regulators of anti-tumor immune response, such as Wnt signaling and TGF beta signaling, are down regulated in both M2 and M3. RNA-Seq also revealed upregulation of alternative signaling pathways such as PI3K/AKT, Notch and Hedgehog signaling in M3 when compared to P1.

### Case study 2:

**Figure 3:**
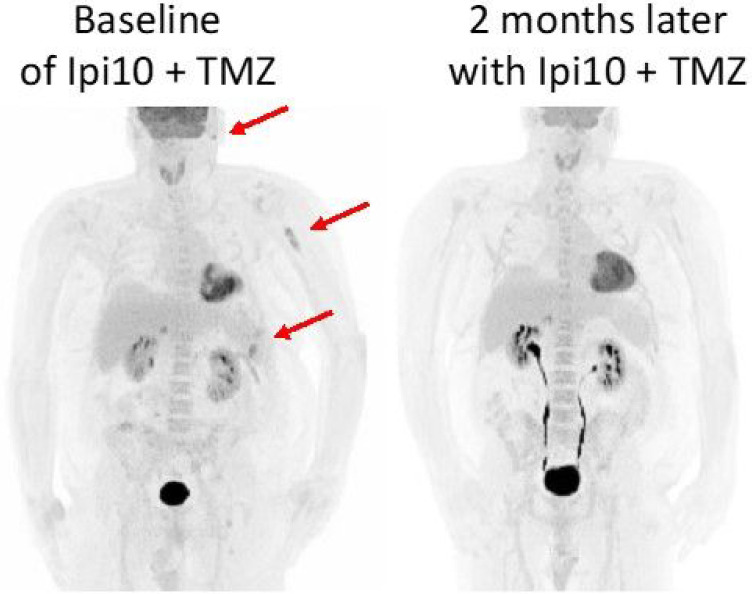
PET CT of patient 10B. PET scan 3 weeks prior to initiation of Ipi10 plus TMZ showed disease progressing with a left preauricular nodule, splenic lesions, left proximal humeral bone marrow space, mesenteric nodes and R level 3 nodes. PET scan 3 months later, after receiving 3 cycles Ipi10 plus TMZ showed dramatic response with complete resolution of lesions in the spleen, mesenteric nodes, left preauricular soft tissue, and left humeral shaft.

A 52 year old male (patient 10B) also responded to high dose Ipi plus TMZ after progressing on multiple lines of therapy. At age 45 he was initially diagnosed with stage IIIC (pT3bN3M0) superficial spreading melanoma to the right forehead. He underwent surgical resection and sentinel lymph node biopsy, as well as right parotidectomy (due to positive sentinel lymph node involvement) and participated in an adjuvant vaccine trial. Three years later he developed a single lung metastasis to the left lower lobe. He underwent wedge resection of lung where this lesion is involved and was started on adjuvant nivolumab. After 5 months of treatment (doses were intermittently held due to toxicity), he developed metastases to the left sided pleura. Next generation sequencing revealed a BRAF V600E mutation, so triple therapy with dabrafenib, trametinib plus pembrolizumab was started. He initially had a partial response to this regimen but 16 months later his melanoma progressed and metastasized to the brain. This was initially managed by SRS to the brain lesion and continued triple therapy, but systemic disease eventually progressed by 7 months. He started Ipi (initially 1mg/kg but increased to 3mg/kg cycles 2–4) plus nivolumab and tolerated all 4 cycles of combination and 2 more doses of monotherapy nivolumab. However, his disease continued to worsen in his CNS with 3 new lesions, and he was started on encorafenib and binimetinib. His disease initially stabilized then progressed both systemically and in the CNS within 10 months. High dose Ipi (10 mg/kg) plus TMZ were started. After 3 cycles, his PET CT revealed partial response and near complete resolution of FDG activity of his disease burden (Figure 3).

### Cohort:

Due to the dramatic response to high dose Ipi plus TMZ in the cases detailed above, we were interested in analyzing the outcomes of other patients with advanced melanoma treated with this combination at our institution. Of the 12 patients with advanced melanoma treated with Ipi plus TMZ, six were treated with high dose Ipi (10 mg/kg), and six were treated with standard dose Ipi (3 mg/kg) (Table 1). Age and BRAF mutations were similar between both groups. All but one patient was previously treated with Ipi. Notable differences between groups include prior exposure to Ipi plus nivolumab. All patients treated with high dose Ipi had prior treatment with Ipi plus nivolumab, whereas only half of patients treated with standard dose Ipi did (Table 1).

This is a very heavily pre-treated patient population with most patients only receiving one dose before moving on to hospice or passing away from disease progression (patients 10E, 10F, 3C, 3D, 3E, 3F). All patients treated with regular dose Ipi plus TMZ had progression of disease as their best response, while two patients treated with high dose Ipi plus TMZ had partial response at the first response evaluation (described in the case section), and a third patient who had a mixed response.

**Figure 4:**
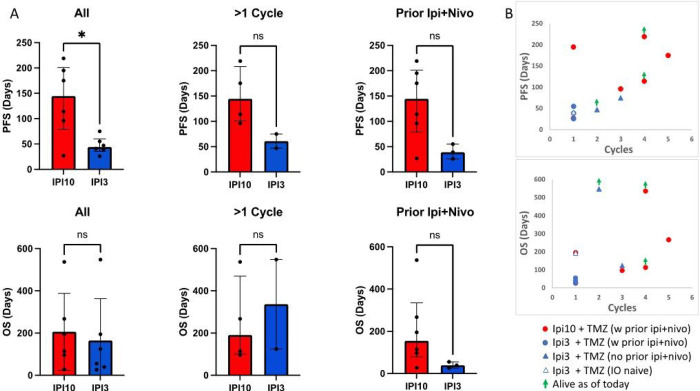
Progression free survival (PFS) and overall survival (OS) in patients treated with Ipi 10 mg/kg plus TMZ vs Ipi 3 mg/kg plus TMZ. A) PFS and OS in all patients (left panel), and patients who had >1 cycle of Ipi 10 mg/kg plus TMZ (middle panel), and who had previous exposure to regular or low dose Ipi plus nivolumab (right panel). B) PFS and OS are plotted against received cycles of Ipi plus TMZ.

At a median follow up of 119 days, patients treated with high dose Ipi plus TMZ had statistically significant longer median progression-free survival (PFS) of 144.5 days (27 – 219) vs 44 days (26 – 75) for standard dose Ipi and TMZ, p=0.04 (Figure 4). Due to the small sample size in each cohort and heterogeneous prior treatment history, no statistically significant differences can be drawn from subgroup analysis; however, there is also a similar trend of longer median PFS for patients who had more than 1 cycle of treatment, 144.5 days (96 – 219) for high dose Ipi plus TMZ vs 61 days (47 – 75) for standard dose Ipi plus TMZ, and in patients who were previously exposed to regular dose Ipi plus nivolumab, 144.5 days (27 – 219) vs 39 days (26 – 55), respectively (Figure 4). There is no statistical difference in terms of median overall survival (OS) but a trend of longer median OS of 154.5 days (27 – 537 days) vs 89.5 days (26 – 548 days) for high and standard dose Ipi plus TMZ. This trend is more evident in patients who were previously exposed to regular dose Ipi plus nivolumab; median OS in the high dose Ipi group was 154.5 days (27 – 537) vs 39 days (26 – 55) of the standard dose Ipi group (Figure 4).

## Discussion

This cohort and case reports highlight the potential for high dose Ipi (10m/kg) plus TMZ in advanced melanoma, including in patients who are heavily pre-treated or have CNS involvement. This study represents a small cohort of patients and is not a randomized controlled trial. However, patients who have progressed on several immunotherapy regimens with poor performance status are often precluded from any clinical trials, and different combinations or dosages are often attempted as a last resort option. Both patients who had a partial response in this report fell into this category and both patients demonstrated remarkable response to Ipi 10 mg/kg plus TMZ, with near complete response of systemic disease and complete remission of CNS disease, despite progressing on all previous regimens including combination Ipi and nivolumab as well as combination immunotherapy with targeted therapy. This confirms a dose-response correlation of Ipi dosing. High-dose Ipi is a reasonable regimen for patients who have already progressed on prior lower dose of Ipi, and the toxicity profile can be successfully managed.

Dosing of anti- PD1 inhibitors does not seem to change efficacy, [20] because these antibodies work by binding to receptors expressed on activated T cells, and greater concentrations do not alter activity. [20, 21] However, the CTLA-4 blockade by Ipi is more nuanced. Previous studies have noted the dose-response relationship of Ipi in advanced melanoma. [6, 22, 23] One study used escalating doses of Ipi on CD4+ T cells of cancer patients. At lower doses, CTLA4 blockade expanded regulatory T cells. Only at the highest dose studied (3 mg/kg) did Ipi expand effector T cells. [24] Furthermore, inhibiting CTLA4 may allow for formation of de novo immune responses in addition to uninhibition of active effector responses. [25] The mechanism and effects Ipi has on T cell activity appears to be influenced by the dose. After progressing on prior Ipi and nivolumab, patients may still develop a robust and durable response to higher dose Ipi. This is highlighted in the cohort, where patients who were previously exposed to low or regular dose of Ipi plus nivolumab responded well to high dose Ipi plus TMZ but had no response to standard dose Ipi plus TMZ, suggesting TMZ is not the determinant of these responses. From this cohort, there is no clear evidence that TMZ has any direct effect in this regimen, although it is reasonable to add TMZ for CNS disease, and more studies are needed to elucidate its effect.

Our molecular investigation by WES and RNA-seq of the tumors in patient 10A provided further insight of potential mechanism of her response to high dose Ipi10 plus TMZ. The patient’s cutaneous primary tumor P1 represents a “cold tumor” with very low activity of the interferon alpha and gamma responses and inflammatory gene sets, and highly active TGF beta and Wnt signaling pathways. This status was completely reversed in M2, a liver metastasis sampled 2 days after the first cycle of low dose Ipi plus nivolumab treatment. There are enriched gene expression and mutations of interferon alpha and gamma response pathways exclusively in the M2 sample, suggesting an enrichment of interferon response and representing a switch to a “hot tumor” after receiving low dose Ipi plus nivolumab treatment. Clinically, the patient did have a mixed response in the liver, and this sampled liver lesion responded to low dose ipi plus nivolumab. However, even after 3 cycles of low dose Ipi plus nivolumab, despite continued response in the liver, her disease progressed to bones and brain with new spine and femur lesions and two new lesions in the left frontal and left occipital lobes. Remarkably, these lesions later responded to high dose Ipi plus TMZ, suggesting a potential “threshold” of Ipi dose for activation of sufficient anti-tumor immune response to control all tumor growth. This threshold could differ in individual patients. For patient 10A, Ipi10 appears to be required for immune control of all her lesions. This provides rationale for higher dose of Ipi after progression on regular or low dose of Ipi-containing regimen. One alternative explanation of her experience is delayed response to her previous low dose Ipi plus nivolumab, and with one case it is hard to prove either way. However, the experience with patient 10B (another partial responder), whose disease progressed after 4 cycles of regular dose Ipi plus nivolumab and 2 more doses of maintenance nivolumab, yet responded to high dose Ipi10 plus TMZ, as well as the significantly prolonged PFS in the high dose Ipi cohort (all patients in this cohort received prior combination of regular or low dose Ipi plus nivolumab), supports the argument that this is not simply a delayed response to prior Ipi plus nivolumab combination, and warrants further investigation.

To our surprise, WES revealed only 12 shared somatic mutations among these tumors. The BRAF V600E mutation is present in all samples, suggesting a common clonal origin, however, RNA-Seq revealed upregulation of alternative signaling pathways such as PI3K/AKT, Notch and Hedgehog signaling in M3 when compared to P1, suggesting potential mechanisms of resistance to interim targeted therapies. The M2 liver metastasis has many more mutations as compared to the primary tumor, possibly due to post- treatment effect as it was taken 2 days after low dose Ipi plus nivolumab was given. Additionally, potential clonal evolution for adaptation to the anatomical liver site may have taken place. M3 is a soft tissue metastasis in the back sampled at disease progression after high dose Ipi plus TMZ (after a 4-month duration of no treatment and high dose steroids for liver toxicity) and at baseline prior to regular Ipi plus nivolumab. M3 has 50 unique mutations when compared to P1, and is enriched with the inflammatory response gene set [26], which may explain why the patient responded to regular Ipi plus nivolumab later.

Anti-PD1 agents were not required for the induction of the response; however, they appear to be important after a response to high dose Ipi to give a durable response. This patient (10A) progressed after high dose Ipi and TMZ were stopped, but responded again to standard dose Ipi plus nivolumab and has a durable response while on maintenance nivolumab only. Since CTLA4 inhibitors may allow formation of *de novo* immune responses, one possible explanation for this outcome is that higher doses may create new antitumor responses. Once established, anti-PD1 agents may work through their primary mechanism of unleashing already established immune responses. [25]

Furthermore, these cases highlight the potential for high dose Ipi and TMZ in brain metastases. A previous study demonstrated a durable response when using this combination in patients with advanced melanoma naïve to immunotherapy, with median overall survival of 24.5 months. However, only 3% of this population had brain metastases. [10] TMZ has been used in treatment of both primary CNS tumors and metastases within the CNS due to its ability to cross the blood brain barrier. [27] The short dosing interval in this regimen (5 days around the Ipi dosing) is mainly to deplete immune suppressive Tregs; however, it is possible it also exerts direct anti-tumor activity towards the brain lesions. One study of 64 patients with advanced melanoma found a 31% response rate to Ipi 10 + TMZ, however only 2 of the patients had brain metastases. [10] Given the poor outcomes in patients with melanoma brain metastases, especially after progression on regular dose of Ipi plus nivolumab, and the dramatic response highlighted in the case report here, further studies are needed to investigate the potential for high-dose Ipi and TMZ in this specific population, as well as in patients with advanced melanoma who have progressed on immune checkpoint inhibitors.

## Figures and Tables

**Table 1:** Baseline characteristics and outcomes of patients treated with Ipi 10 mg/kg + TMZ (orange) and Ipi 3 mg/kg + TMZ (blue).

Pt ID	Gender	Age at C1D1	Ipilimumab mg/kg	TMZ 200 mg/m2 D1-D4	Melanoma Subtype	BRAF	Prior Therapies	No of Cycles	Best Response	PFS	OS	Alive	Next Lines of Therapies	irAE	Grade	HD steroids?
**10A**	F	30	10	Y	Nodular	V600E	neoadj pembro, D+T+P, ipi1+nivo3	4	PR	219	537	Y	ipi3+nivo1	Hepatitis	3	Y
**10B**	M	52	10	Y	Superficial Spreading	V600E	adj nivo, D+T+P, ipi3+nivo1, E+B	4	PR	114	114	Y	None (continued response)	Rash	3	Y
**10C**	F	48	10	Y	Acral	WT	ipi3+nivo1, palbociclib+nivo	5	Mixed Response	175	267	N	lenvatinib + pembro	Diarrhea	2	Y
**10D**	M	35	10	Y	Non-Acral Cutaneous	V600E	ipi3, pembro, ipi3+nivo1, D+T+P	3	PD	96	96	N	None	PE, SOB	3	Y (for brain mets)
**10E**	F	67	10	Y	Acral	WT	neoadj nivo, ipi3+nivo1, microbiome+nivo, lenvatinib+pembro	1	PD	195	195	N	None	Diarrhea	3	Y
**10F**	M	66	10	Y	Nodular	V600E	adj nivo, D+T, E+B, ipi3+nivo1	1	PD	27	27	N	None	No	0	N
**3A**	M	47	3	Y	Non-Acral Cutaneous	V600E	ipi3, IL-2, dabra, pembro, D+T	3	PD	75	124	N	carbo	No	0	N
**3B**	M	54	3	Y	Nodular	V600K	ipi3, D+T, neoadj TVEC, adj nivo, D+T+P	2	PD	47	548	Y	carbo+taxol, lenvatinib+pembro, rela+nivo	Eye muscle inflammation	3	Y
**3C**	M	69	3	Y	Acral	WT	TVEC+pembro, ipi3+Cavatak, ipi3+nivo1	1	PD	26	26	N	None	No	0	N
**3D**	M	45	3	Y	Non-Acral Cutaneous	V600E	ipi3+nivo1, nivo, D+T, pembro, D+T+P	1	PD	55	55	N	None	No	0	N
**3E**	F	21	3	Y	Nodular	V600E	adj D+T, neoadj pembro, ipi3+nivo1, TVEC+pembro, TMZ 75mg/kg daily	1	PD	39	39	N	None	No	0	N
**3F**	M	52	3	Y	Choroidal	WT	MEK+AKT inhibitors	1	PD	41	194	N	pembro, disulfiram/zinc	No	0	N

## Data Availability

No publically available data sets were used, however workflows used were publicly available and detailed in the methods section. The alignment, variant calling, variant annotations were integrated in containerized Snakemake workflows that are publicly available at https://github.com/HuntsmanCancerInstitute/Workflows
